# Disease-specificity in correlations between speech audiometry, hearing-related quality of life, and varying calculation methods for pure tone average in middle ear diseases

**DOI:** 10.1007/s00405-026-10335-4

**Published:** 2026-06-01

**Authors:** Ivo Grueninger, Julia van de Loo, Sara Alekuzei, Kevin Karl Hansen, Aviva Henze, Jennifer Spiegel, Daniel Polterauer, Maike Neuling, Hans N. C. Eckel, Marcel Mayer, Jan Christoffer Luers, Jens Peter Klußmann, Martin Canis, Joachim Müller, Martin Walger, Tobias Rader, Kariem Sharaf

**Affiliations:** 1 Department of Otorhinolaryngology, Helios Hospital Berlin-Buch, Schwanebecker Chaussee 50, 13125 Berlin, Germany; 2 Department of Otorhinolaryngology, University Hospital, LMU Munich, Marchioninistrasse 15, 81377 Munich, Germany; 3 Medical Faculty, Department of Otorhinolaryngology, Head and Neck Surgery, University of Cologne, Kerpener Str. 62, D-50937, 50924 Cologne, Germany; 4 Department of Otorhinolaryngology, Head and Neck Surgery, University Hospital, Elfriede-Aulhorn-Strasse 5, 72076 Tübingen, Germany

**Keywords:** Conductive hearing loss, Mixed hearing loss, Otitis media, Otosclerosis, Pure tone average, Quality of life, AAO-HNS guideline

## Abstract

**Purpose:**

This study examined whether different methods for calculating pure tone average (PTA) yield clinically relevant differences in correlations with speech audiometry and hearing-related quality of life (QoL) in conductive and mixed hearing loss. Additionally, we investigated whether these associations vary among distinct middle ear pathologies: otosclerosis, acute otitis media (AOM) and chronic otitis media (COM).

**Methods:**

In an ambispective cohort study at a tertiary referral center, 220 ears were analyzed. 194 ears were collected retrospectively. 26 patients were recruited prospectively. Inclusion criteria were adult age, confirmed diagnosis, near-normal bone conduction and conductive hearing loss without prior middle ear surgery. Pure-tone and Freiburg speech audiometry were analyzed along with the SSQ12 questionnaire in prospective patients. Multiple air conduction (AC) and air–bone gap (ABG) - PTA configurations were compared using linear regression and ANCOVA.

**Results:**

Across all cohorts, PTA values strongly correlated with speech recognition at 65 dB SPL and with 50% number recognition thresholds (R^2^ up to 0.77). No clinically relevant differences were found between 4-frequency-PTAs using 3 kHz vs. 4 kHz. However, disease-specific patterns emerged: otosclerosis patients showed consistently poorer word recognition than AOM or COM patients at comparable PTA levels. QoL outcomes correlated significantly with AC-based PTAs but not with ABG-based PTAs or speech results.

**Conclusions:**

PTA calculation methods yield comparable correlations with speech understanding; inclusion of 3 or 4 kHz does not alter results meaningfully. Disease-specific discrepancies underscore the need for nuanced interpretation of PTA–speech relationships and support integrating both PTA and QoL measures in patient assessment. The study was conducted under clinical trial registration number DRKS00037758.

**Supplementary Information:**

The online version contains supplementary material available at https://doi.org/10.1007/s00405-026-10335-4.

## Introduction

2025 marks the 30th anniversary of the American Academy of Otolaryngology–Head and Neck Surgery (AAO-HNS) publishing guidelines outlining minimum standards for reporting hearing outcomes in clinical studies on conductive hearing loss (CHL) [1]. Pure-tone and speech audiometry are still among the most fundamental routine diagnostic tools in otology, providing the basis for determining therapy indications and evaluating treatment success.

The 1995 publication aimed to standardize reporting to improve comparability and thereby facilitate the creation of data pools for meta-analyses [1]. For the pure tone average (PTA), the frequencies 0.5, 1, 2, and 3 kHz were defined.

The AAO-HNS guidelines have since been the subject of extensive discussion and criticism, particularly regarding the selection of frequencies used for calculating the PTA [2–5]. Later revisions of the AAO-HNS recommendations introduced a core outcome set (COS) consisting of the PTA and the word recognition score (WRS). The selection of test frequencies for the PTA remained unchanged, although averaging the value for 3 kHz from the 2 and 4 kHz thresholds was explicitly permitted [6]. Since 2012, the AAO-HNS recommendations apply not only to CHL but to all types of hearing loss.

Despite the stated goal of standardization to improve comparability, the AAO-HNS guidelines have been only partially or not at all implemented by the otologic community [7]. A recent review clearly illustrates how heterogeneous and thus poorly comparable the reported outcome parameters remain in otologic studies on mixed hearing loss (MHL) and CHL [8]. In particular, standardized reporting of a COS as demanded by the AAO-HNS since 1995/2012 is still lacking.

In our research population, the PTA is traditionally and still most commonly calculated using the frequencies 0.5, 1, 2, and 4 kHz, thereby deviating from the AAO-HNS recommendation. A recent survey also demonstrates significant heterogeneity in routine audiological diagnostics within this population [9].

In addition, patient-reported outcome measures (PROMs), particularly those assessing disease-specific quality of life (QoL), are increasingly being collected and reported. However, national or international recommendations for the selection of PROMs are missing.

The continuing lack of standardization not only hampers the comparability of study results but also complicates patient counseling in everyday clinical practice. The value of the PTA for the daily practice is further complicated by the reported discrepancies in the correlation between PTA and speech understanding for different middle ear pathologies leading to CHL or MHL [10].

Our research questions were whether we could a) observe clinically significant differences between varying PTA calculation methods and their correlation to speech understanding and hearing-related QoL in real-world data from the daily practice and b) observe differences for different underlying middle ear pathologies primarily leading to CHL or MHL. Eventual findings are supposed to enable the clinician to evaluate reported hearing outcomes more effectively from studies, enabling them to provide better informed advice and counseling to individual patients in the daily clinical practice.

## Material and methods

### Case selection

For this explorative, observational, combined retrospective/prospective single-center study, we identified 220 ears from an otology practice of a tertiary referral center. To assemble disease-specific retrospective cohorts, potential cases were identified by searching for International Classification of Diseases version 10 (ICD-10) codes for otosclerosis, acute otitis media (AOM), and chronic otitis media (COM) in the clinical information system (SAP® 8 R/3 resp. S/4 HANA, Walldorf, Germany). In parallel, we recruited a prospective cohort of consecutive eligible patients willing to participate, from a specialized otology outpatient clinic.

A thorough description of the case selection process is offered in the Supplemental material (Supplemental Methods - Detailed case selection).

Eligibility criteria were 1. adult age (≥ 18a), 2. ENT-specialist proven diagnosis of a) otosclerosis, b) AOM or c) COM on one or both ears, 3. no previous middle ear surgery (except for myringotomy and/or grommet insertion), 4. available data on both pure tone and speech audiometry, 5. normal or moderately altered BC (≤ 30 dBHL in every tested frequency), and 6. CHL of ≥ 10 dB in at least three different tested frequencies.

The basic patient characteristics of all remaining ears of the retrospective cohorts and the prospective cohort are depicted in Table 1.

**Table 1:** Baseline characteristics *n* (%) or mean ± SD

	Total	C1	C2	C3	C4
*n* =	220 (100.0)	26 (11.8)	76 (34.5)	32 (14.5)	86 (39.1)
Gender (female)	132 (60.0)	10 (38.4)	51 (67.1)	20 (62.5)	51 (59.3)
Mean age (years)	39.0 ± 4.7	41.1 ± 8.9	42.2 ± 12.3	41.8 ± 17.1	34.5 ± 16.1
65 dB monosyllables (%)	78.4 ± 24.5	66.2 ± 34.2	75.2 ± 25.4	86.2 ± 15.4	82.4 ± 20.5
80 dB monosyllables (%)	92.7 ± 14.7	92.1 ± 16.3	89.4 ± 15.3	94.6 ± 13.7	95.2 ± 12.9
50% number recognition (dB)	46.9 ± 11.5	48.2 ± 11.7	51.6 ± 10.7	42.7 ± 9.4	44.2 ± 11.6
ABG average 0.5,1,2,3 kHz	17.5 ± 8.5	18.3 ± 9.0	18.5 ± 8.5	13.7 ± 7.5	17.9 ± 8.4
ABG average 0.5,1,2,4 kHz	18.9 ± 8.4	19.5 ± 8.6	20.2 ± 8.4	15.4 ± 7.3	18.9 ± 8.4
Subgroups in C1					
COM w Chole	6 (23.1)				
COM w/o Chole	3 (11.5)				
Otosclerosis	16 (61.5)				
other	1 (3.8)				
Subgroups in C4					
w Chole	18 (8.2)				
w/o Chole	68 (30.9)				

*SD* standard deviation, *dB* decibels, *ABG* air bone gap, *C1* cohort 1 (prospective mixed), *C2* cohort 2 (otosclerosis), *C3* cohort 3 (otitis media acuta), *C4* cohort 4 (otitis media chronica)

### Data collection

Basic patient characteristics, as well as diagnosis related data and audiometric measures of the surgical cases were obtained. For evaluation, data were transferred to a clinical database (ENTstatistics, INNOFORCE, Ruggell, Liechtenstein).

### Audiometric measurements

Pure tone audiometry data were collected at frequencies 0.25, 0.5, 1, 1.5, 2, 3, 4, and 6 kHz for bone conduction (BC) and additional frequencies 0.125, and 8 kHz for air conduction (AC). We used the Freiburg Speech Test for monosyllabic word recognition at 65 and 80 dB SPL as well as the 50% number recognition threshold for polysyllable numbers in dB. To assess for hearing-related QoL the Speech, Spatial and Qualities of Hearing Scale – Short Form (SSQ12) was used [11].

For the retrospective cohorts, data were collected and included as much as the aforementioned data from pure tone and speech audiometry were available. In few retrospective cases, audiometric data for 3 kHz was missing and was therefore imputated as described previously [12]. Regarding speech audiometry, in cases, in which monosyllabic word recognition at 80 dB SPL and 50% number recognition had not been directly measured, it was imputated accordingly [13]. For 50% number recognition missing data was imputated using a linear interpolation between two measurements (one data point with number-recognition > 50% and one data point with number-recognition < 50%). Missing data points for monosyllabic word recognition at 80 dB SPL were set to 100% if measurements for levels < 80 dB SPL yielded ≥ 95% word recognition in the same case. This approach allowed for a consistent estimation of these values in cases lacking direct measurement [14].

Audiometric testing was performed using an AT1000 audiometer (Auritec Medizindiagnostische Systeme, Hamburg, Germany) with DT48A headphones (Beyerdynamic, Heilbronn, Germany) and a B71 bone conduction vibrator (Radioear, Middelfart, Denmark). The measurements were collected using standardized audiometric procedures in compliance with ISO norm 8253–1:2010.

The following PTA calculation methods were included:

- Air conduction (AC) and air bone gap (ABG) averages at 0.5, 1, 2, 3 kHz
- AC and ABG averages at 0.5, 1, 2, 4 kHz
- AC and ABG averages at 0.5, 1, 2, 3, 4 kHz
- Extended AC averages at 0.125, 0.25, 0.5, 1, 2, 3, 4, 5, 8 kHz
- Extended ABG averages at 0.25, 0.5, 1, 2, 3, 4, 6 kHz

To analyze whether taking low and high frequency data into account yields better correlation to WRS and hearing-related QoL, extended PTAs were included in the exploratory analysis as well.

### Statistical analysis

All statistical analyses were conducted using IBM SPSS Statistics for Mac, version 29 (Amonk, New York, US). Descriptive statistics were reported as absolute numbers and percentages for categorical variables, and as means with standard deviations (mean ± SD) for continuous variables. Differences in QoL between groups were assessed with independent sample t-Test. To assess the relationships between audiometric parameters and speech audiometry as well as QoL outcomes, linear regression analyses were performed and conducted separately for each cohort. To examine differences in correlation strength between cohorts, regression slopes were statistically compared using interaction terms in an analysis of covariance (ANCOVA) model. These comparisons tested for significant differences in the linear relationship between PTA measures and outcome variables across the disease groups.

All p-values are reported two-sided. Results with *p* < 0.05 were considered statistically significant. Due to the explorative character of the study no p-value adjustment for multiple testing was considered. When appropriate, R^2^ values were reported to reflect the proportion of variance explained by the respective model. Differences in R^2^ values ≥ 0.05 were considered as clinically relevant.

### Conflict of interest and ethics statement

All clinical data were obtained and analyzed based on the approval by the ethics committee of the university hospital LMU Munich (IRB approval number 21–0048) and in compliance with the Declaration of Helsinki. The study was conducted under clinical trial registration number DRKS00037758 (https://www.drks.de/DRKS00037758).

## Results

**Fig. 1:**
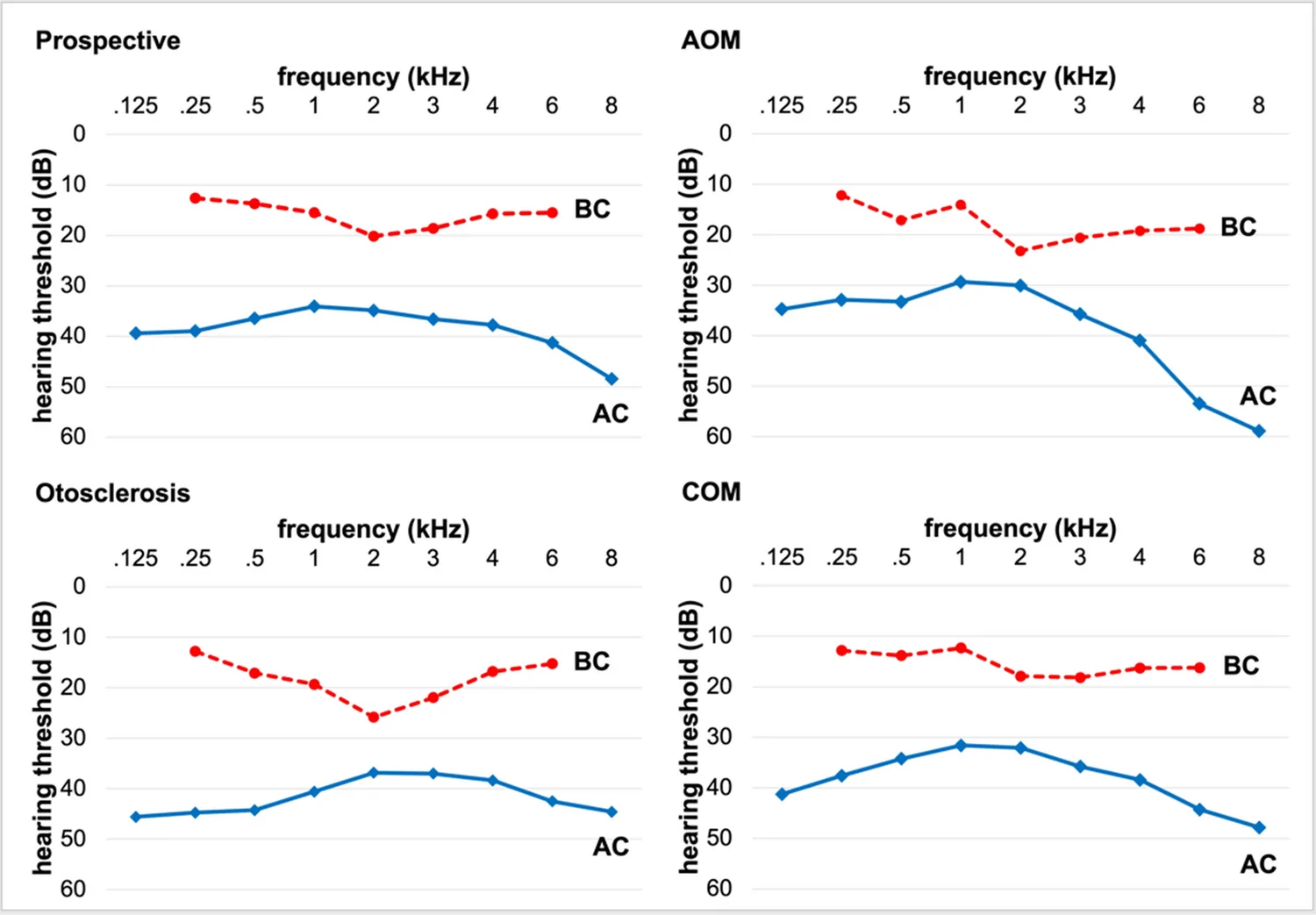
Pure tone audiometry averages in 4 cohorts. dB = decibels; kHz = kilohertz; red line = bone conduction; blue line = air conduction, AOM = acute otitis media; COM = chronic otitis media

In total, 220 ears were included in this study. 60% were female and had a mean age of 39.0 ± 14.7 years. The prospective cohort (C1, *n* = 26) included patients with various middle ear pathologies (61.5% otosclerosis, 23.1% COM with and 11.5% COM without cholesteatoma), while the retrospective cohorts consisted of 76 otosclerosis ears (C2), 32 AOM ears (C3) and 86 COM ears (C4, 18 with and 68 without cholesteatoma). Patient characteristics are depicted in Table 1 and the average audiogram (AC and BC) for each cohort is demonstrated in Fig. 1. In the retrospective cohorts, imputation of missing data was necessary for PTA (missing data point for 3 kHz) in 3.6% of cases (otosclerosis: 3.9%, COM: 2.1%, AOM: 6.3%) and for speech audiometry in 67.3% of cases for the 50%-recognition threshold for polysyllable numbers (otosclerosis: 65.5%, COM: 71.8%, AOM: 58.6%) and 37.7% of cases for monosyllabic word recognition at 80 dB SPL (otosclerosis: 25.4%, COM: 46.7%, AOM: 42.9%), while no imputation was needed in the prospectively recruited cohort and for monosyllabic word recognition at 65 dB SPL in all cases.

### Comparison of PTA calculation methods across different middle ear diseases

**Fig. 2:**
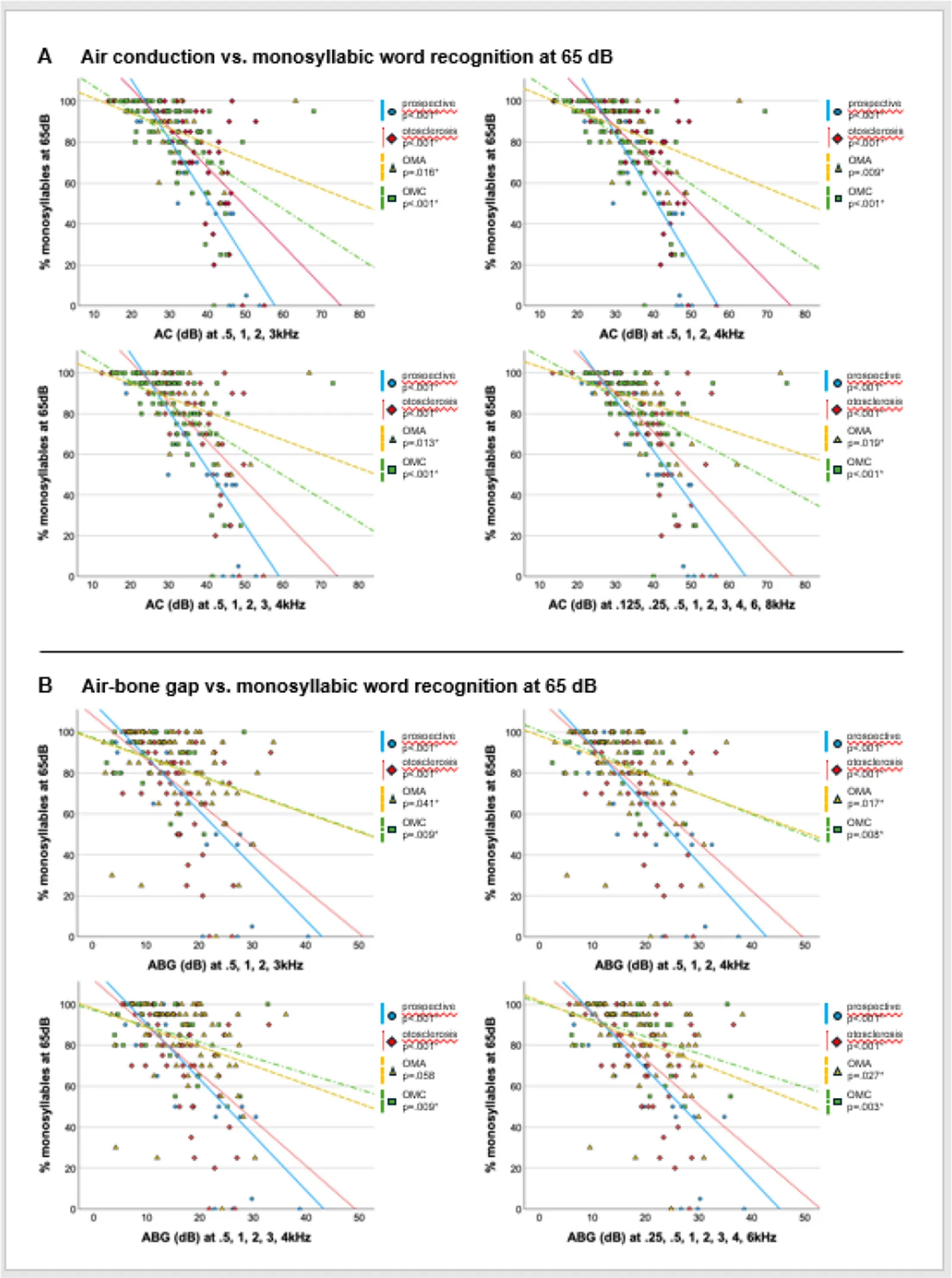

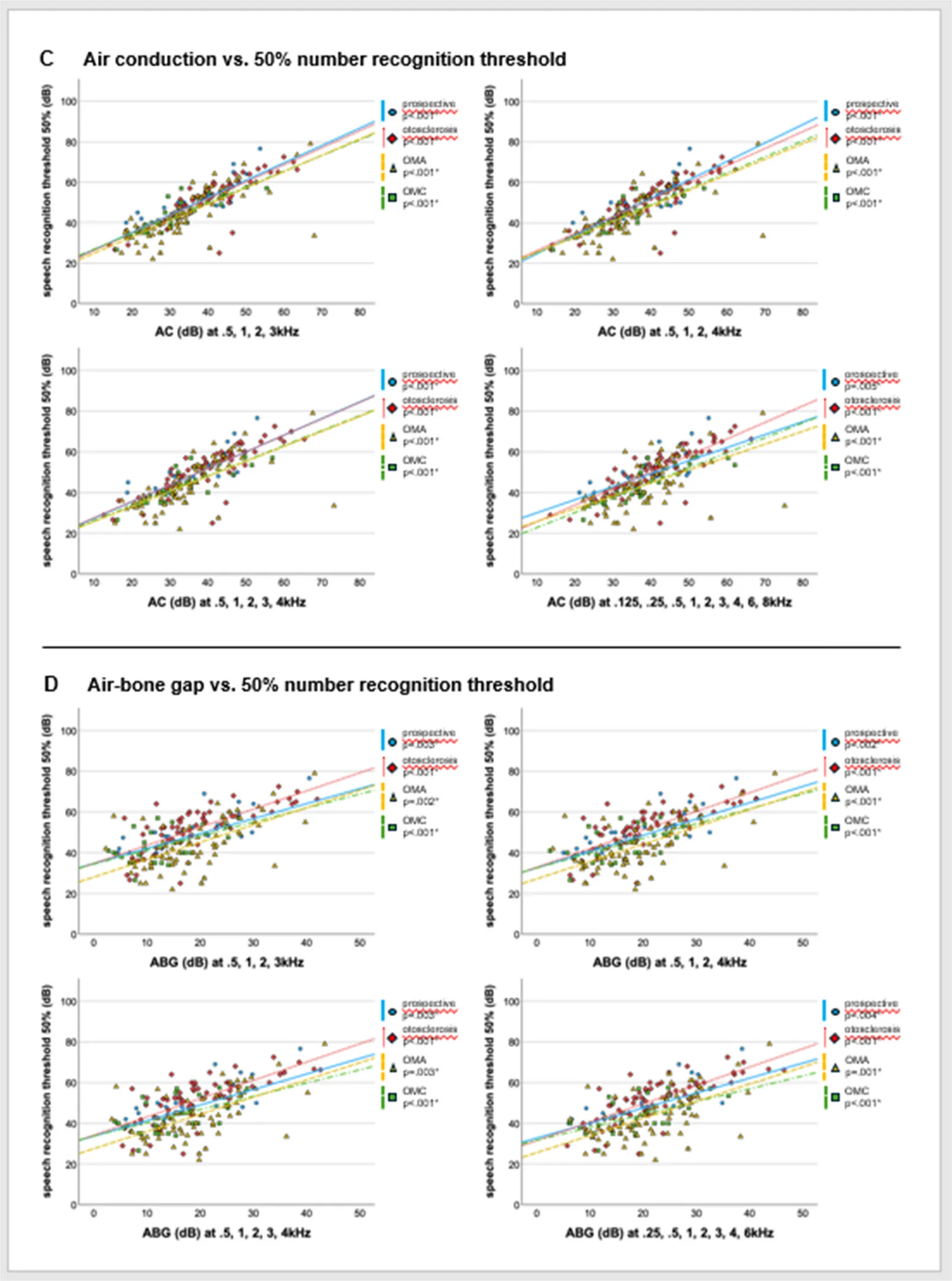
Correlation between pure tone hearing test results and speech (linear regression). For the four cohorts, correlations are reported between air conduction (**A**, **C**) or air–bone gap (**B**, **D**), and monosyllabic word recognition at 65 dB (Freiburg speech test, **A**, **B**) or the 50% number recognition threshold (**C**, **D**). dB = decibels; kHz = kilohertz; red line = bone conduction; blue line = air conduction, AOM = acute otitis media; COM = chronic otitis media

In the prospective cohort, representing non-acute cases from a specialized otology outpatient clinic, all tested PTA configurations showed highly significant correlations with both 65 dB monosyllable recognition and 50% number recognition thresholds (all *p* < 0.005, Fig. 2, supp. Table S1). R^2^ values for AC measures ranged from 0.52 to 0.7 (65 dB) and from 0.28 to 0.52 (50% number) with the lowest R^2^ values for PTA configuration using all available frequencies. Even ABG-based metrics demonstrated moderately strong correlations (R^2^ = 0.47–0.51 and 0.3–0.34, respectively).

These findings were consistently replicated in all retrospective cohorts and except for one tested correlation (65 dB monosyllables and ABG-PTA 0.5, 1, 2, 3, 4 kHz for the AOM subcohort; *p* = 0.058), all types of PTA also correlated significantly with 65 dB monosyllables and 50% number recognition. There were some variations in correlation strength depending on the underlying disease. For instance, in otosclerosis, AC-PTA showed strong associations with speech outcomes (e.g., AC at 0.5, 1, 2, 4 kHz: R^2^ = 0.45 for 65 dB monosyllables and R^2^ = 0.71 for 50% number recognition) while in AOM, correlations were weaker for 65 dB monosyllables (e.g., R^2^ = 0.2–0.24), but again high for 50% number thresholds (R^2^ up to 0.77). COM (C4) showed moderate-to-strong associations for both outcomes (R^2^ = 0.22–0.3 for 65 dB; R^2^ = 0.34–0.57 for 50% number).

Overall, there were only slight differences in correlation strengths (R^2^ values) between different PTA calculation methods within each correlated set (cohort, AC or ABG, 65 dB monosyllables or 50% number thresholds; compare Table S1).

### Comparison of PTA calculation methods between different middle ear diseases

To assess whether these correlation differences were clinically meaningful between diseases as well as disease-specific cohorts compared to a mixed cohort from a specialized otology outpatient clinic, we compared regression slopes pairwise (Tables 2 and 3, Fig. 2): For 65 dB monosyllables, significant or tendentially significant differences in correlation strengths were found between the otosclerosis cohort and both otitis media cohorts as well as the prospective cohort and all retrospective cohorts for most PTA variants (e.g., AC 0.5, 1, 2, 4 kHz: *p* < 0.001 for prospective vs. COM and vs. AOM; *p* = 0.022 vs. otosclerosis), while no significant differences in correlation strengths were found between both otitis media cohorts (Table 2, Fig. 2). On the other hand, there was no significant difference between the prospective cohort and otosclerosis using the air–bone gap as PTA across different frequencies (Table 2, Fig. 2). Interestingly, there were no differences at all when comparing PTA to 50% number recognition between the cohorts, where no significant slope differences were detected (Table 3, Fig. 2).

**Table 2:** Comparison of correlations between groups PTA to 65 dB monosyllables

	**prospective vs. otosclerosis**	**prospective vs. AOM**	**prospective vs. COM**
air conduction 0.5,1,2,3 kHz	*p* = 0.046*	*p* = 0.001*	*p* < 0.001*
air conduction 0.5,1,2,4 kHz	*p* = 0.022*	*p* < 0.001*	*p* < 0.001*
air conduction 0.5,1,2,3,4 kHz	*p* = 0.087	*p* < 0.001*	*p* < 0.001*
0.125,0.25,0.5,1,2,3,4,6,8 kHz	*p* = 0.256	*p* < 0.001*	*p* < 0.002*
air bone gap 0.5,1,2,3 kHz	*p *= 0.470	*p* = 0.021*	*p* = 0.004*
air bone gap 0.5,1,2,4 kHz	*p* = 0.486	*p* = 0.015*	*p* = 0.003*
air bone gap 0.5,1,2,3,4 kHz	*p* = 0.547	*p* = 0.010*	*p* = 0.005*
0.25,0.5,1,2,3,4,6 kHz	*p* = 0.498	*p* = 0.008*	*p* = 0.005*
	**AOM vs. COM**	**AOM vs. otosclerosis**	**COM vs. otosclerosis**
air conduction 0.5,1,2,3 kHz	*p* = 0.247	*p* = 0.011*	*p* = 0.056
air conduction 0.5,1,2,4 kHz	*p* = 0.222	*p* = 0.010*	*p* = 0.069
air conduction 0.5,1,2,3,4 kHz	*p* = 0.224	*p* = 0.003*	*p* = 0.029*
0.125,0.25,0.5,1,2,3,4,6,8 kHz	*p* = 0.306	*p* = 0.002*	*p* = 0.014*
air bone gap 0.5,1,2,3 kHz	*p* = 0.996	*p* = 0.107	*p* = 0.045*
air bone gap 0.5,1,2,4 kHz	*p* = 0.903	*p* = 0.057	*p* = 0.016*
air bone gap 0.5,1,2,3,4 kHz	*p* = 0.797	*p* = 0.030*	*p* = 0.023*
0.25,0.5,1,2,3,4,6 kHz	*p* = 0.735	*p* = 0.035*	*p* = 0.036*

*kHz* kilohertz, *dB* decibels, *AOM* otitis media acuta, *COM* otitis media chronica

**Table 3:** Comparison of correlations between groups PTA to 50% number recognition

	**prospective vs. otosclerosis**	**prospective vs. AOM**	**prospective vs. COM**
air conduction 0.5,1,2,3 kHz	*p* = 0.929	*p* = 0.645	*p* = 0.773
air conduction 0.5,1,2,4 kHz	*p* = 0.666	*p* = 0.778	*p* = 0.470
air conduction 0.5,1,2,3,4 kHz	*p* = 0.964	*p* = 0.687	*p* = 0.688
0.125,0.25,0.5,1,2,3,4,6,8 kHz	*p* = 0.330	*p* = 0.669	*p* = 0.977
air bone gap 0.5,1,2,3 kHz	*p* = 0.470	*p* = 0.879	*p* = 0.625
air bone gap 0.5,1,2,4 kHz	*p* = 0.596	*p* = 0.812	*p* = 0.835
air bone gap 0.5,1,2,3,4 kHz	*p* = 0.539	*p* = 0.719	*p* = 0.744
0.25,0.5,1,2,3,4,6 kHz	*p* = 0.446	*p* = 0.722	*p* = 0.683
	**AOM vs. COM**	**AOM vs. otosclerosis**	**COM vs. otosclerosis**
air conduction 0.5,1,2,3 kHz	*p* = 0.825	*p* = 0.566	*p* = 0.727
air conduction 0.5,1,2,4 kHz	*p* = 0.913	*p* = 0.667	*p* = 0.522
air conduction 0.5,1,2,3,4 kHz	*p* = 0.990	*p* = 0.565	*p* = 0.535
0.125,0.25,0.5,1,2,3,4,6,8 kHz	*p* = 0.589	*p* = 0.555	*p* = 0.166
air bone gap 0.5,1,2,3 kHz	*p* = 0.533	*p* = 0.374	*p* = 0.825
air bone gap 0.5,1,2,4 kHz	*p* = 0.635	*p* = 0.393	*p* = 0.698
air bone gap 0.5,1,2,3,4 kHz	*p* = 0.466	*p* = 0.269	*p* = 0.760
0.25,0.5,1,2,3,4,6 kHz	*p* = 0.408	*p* = 0.192	*p* = 0.718

*kHz* kilohertz, *dB* decibels, *AOM* otitis media acuta, *COM* otitis media chronica

### Relationship between audiometric measures and quality of life (QoL)

Finally, we explored how subjective hearing measures relate to subjective hearing-related QoL, as captured by the SSQ12. Notably, only PTA - especially AC-based averages at core and extended frequencies - showed significant associations with QoL scores (Table 4): almost all types of PTA calculations based on AC were significantly correlated with QoL in all subdomains. An exception was the subgroup of q. 6–8 from the SSQ12 questionnaire, which reflect spatial hearing. Interestingly, there was no statistical association between the different methods of calculating PTA using the ABG and the questionnaire subgroups or the overall score. Moreover, none of the speech audiometry parameters (65 dB or 80 dB monosyllables, nor 50% number recognition thresholds) showed significant association with subjective QoL (Table 4). There was no significant difference in QoL depending on whether patients had unilateral or bilateral middle ear pathology, regarding speech in noise (items 1–4, *p* = 0.635), spatial hearing (items 6–8, *p* = 0.518) or overall-hearing related QoL (items 1–12, *p* = 0.649).

**Table 4:** Correlation between hearing test results and quality of life (linear regression)

	q1 - 5	q1 - 4	q2 - 5	q6 - 8	q9 - 12	q1 - 12
65 dB monosyllables	*p* = 0.180	*p* = 0.315	*p* = 0.293	*p* = 0.413	*p* = 0.487	*p* = 0.303
80 dB monosyllables	*p* = 0.580	*p* = 0.867	*p* = 0.802	*p* = 0.757	*p* = 0.833	*p* = 0.849
50% number recognition	*p* = 0.086	*p* = 0.195	*p* = 0.172	*p* = 0.326	*p* = 0.337	*p* = 0.176
air conduction 0.5,1,2,3 kHz	*p* = 0.027*	*p* = 0.048*	*p* = 0.041*	*p* = 0.165	*p* = 0.043*	*p* = 0.039*
air conduction 0.5,1,2,4 kHz	*p* = 0.016*	*p* = 0.035*	*p* = 0.023*	*p* = 0.231	*p* = 0.038*	*p* = 0.036*
air conduction 0.5,1,2,3,4 kHz	*p* = 0.016*	*p* = 0.031*	*p* = 0.023*	*p* = 0.211	*p* = 0.029*	*p* = 0.031*
0.125,0.25,0.5,1,2,3,4,6,8 kHz	*p* = 0.035*	*p* = 0.050*	*p* = 0.056	*p* = 0.413	*p* = 0.071	*p* = 0.075
air bone gap 0.5,1,2,3 kHz	*p* = 0.145	*p* = 0.166	*p* = 0.174	*p* = 0.178	*p* = 0.103	*p* = 0.099
air bone gap 0.5,1,2,4 kHz	*p* = 0.124	*p* = 0.151	*p* = 0.145	*p* = 0.134	*p* = 0.103	*p* = 0.081
air bone gap 0.5,1,2,3,4 kHz	*p* = 0.104	*p* = 0.120	*p* = 0.122	*p* = 0.147	*p* = 0.078	*p* = 0.071
0.25,0.5,1,2,3,4,6 kHz	*p* = 0.089	*p* = 0.095	*p* = 0.117	*p* = 0.146	*p* = 0.084	*p* = 0.068

*kHz* kilohertz, *q* questions. All reported p-values correspond to the sums of the indicated questions (q) from the SSQ12 questionnaire

## Discussion

This study consistently demonstrated strong correlations between various PTA-calculation methods and speech recognition outcomes - specifically 65 dB monosyllabic scores and 50% number recognition thresholds - across all examined middle ear pathologies (otosclerosis, AOM, and COM). Notably, we found no clinically relevant differences between commonly used 4-frequency PTA calculation methods that include either 3 kHz or 4 kHz in terms of their correlation with speech audiometry outcomes (Table S1). This finding was consistent across all analyzed middle ear pathologies. Moreover, extended PTA calculations (e.g., 0.125–8 kHz for AC, 0.5–6 kHz for ABG) did not provide stronger correlations with speech audiometry compared to standard methods.

Following the initial AAO-HNS guidelines of 1995, Goldenberg et al. evaluated various PTA calculation strategies in 550 patients with chronic ear conditions and found minimal differences in postoperative AC-PTAs among frequency combinations including up to 4 kHz [5]. These findings align well with our data. In a subsequent work on stapes surgery outcomes, the same group reported that - unlike in chronic ear surgery - success rates varied based on PTA calculation method, with lower rates observed when 4 kHz was included in the 4-frequency average rather than 3 kHz [2]. Similar results were reported by de Bruijn et al., who evaluated 451 stapes surgeries and found the highest success rates using the traditional 3-frequency PTA (0.5, 1, 2 kHz). While PTA calculation had limited impact on postoperative ABG levels, AC gain at 0.5, 1, 2, and 4 kHz was found to correlate best with speech audiometry gains [4]. In another study, v. Gierke et al. analyzed 504 ossiculoplasties and concluded that PTAs emphasizing lower frequencies (0.5–2 kHz) produced more favorable postoperative results compared to high-frequency-inclusive methods, with the poorest outcomes for a 5-frequency PTA including 6 kHz [15].

In our study, we did not investigate 2- or 3-frequency PTAs limited to low frequencies, given the widespread clinical use of broader PTA calculations including higher frequencies. The modest differences in outcomes between 4-frequency PTAs using 3 kHz versus 4 kHz in previous studies do not contradict our results. Our focus was not on surgical success rates - often measured by ABG closure - but rather on how different PTA calculation methods relate to speech-understanding and hearing-related QoL in surgery-naive ears.

When comparing middle ear pathologies, we found strong correlations between PTA and word recognition scores (WRS) measured via the Freiburg Speech Test in all groups (Table S1). However, speech understanding in otosclerosis was consistently poorer than in AOM or COM, despite similar PTA values across calculation methods (Fig. 2, Tables 2 and 3). This trend was evident across both retrospective and prospective cohorts and applied to PTAs calculated using various frequency combinations for both AC and ABG. Statistically significant differences were identified between otosclerosis and COM (ABG-based PTAs) and between otosclerosis and AOM (AC-based PTAs), particularly when using 4 kHz rather than 3 kHz for the PTA calculation. Extended PTA calculations (5- or 9-frequency AC PTAs; 5- or 7-frequency ABG PTAs) also revealed significant differences in WRS–PTA correlations between otosclerosis and both AOM and COM (Table 2).

Interestingly, this disease-specific impairment in speech recognition was not evident when analyzing 50% number recognition thresholds for numbers (Table 3), suggesting that this metric may be less sensitive to pathology-specific differences - likely due to its emphasis on lower frequencies avoiding the Carhart notch as well as the high frequency region > 2 kHz, which is mostly affected in patients with AOM and COM [16]. The 50% number recognition threshold emerged as a robust, disease-independent correlate of PTA at lower frequencies.

Middle ear mechanics play an important role in disease-specificity. In otosclerosis, based on the localization of the otosclerotic focus, different types of hearing loss evolve, usually a conductive or mixed hearing loss starting mostly in the low frequency range and rarely an isolated sensorineural type [17, 18]. In particular, the Carhart notch is most pronounced around 2 kHz (especially after complete fixation of the stapes footplate). The Carhart effect in stapes surgery is known to influence audiometric outcome parameters, neglecting it can result in overoptimistic reporting of surgical success [16, 19–22]. Carhart effect has also been identified in otitis media, especially in AOM with effusion but also in COM and may reflect a share of cases of otitis media with an ossicle fixation of some extent [23–25]. The precise influence of different kinds of middle ear impairment (e.g. stiffening as in otosclerosis or reduction of compliance as in middle ear effusion) and resulting impairment of middle ear transfer function has been examined by numerous groups in experimental studies giving insights into the resonance frequencies of the middle ear and specific effects for different conditions [26–28]. Beyond the pure magnitude of the conductive component, alterations in middle ear mechanics may also influence speech perception. In otosclerosis, fixation of the stapes footplate increases stiffness of the ossicular chain and alters the middle ear transfer function, resulting in frequency-specific distortions of sound transmission rather than a simple attenuation across frequencies [27]. Such mechanical changes may affect the fidelity with which complex acoustic signals, such as speech, are transmitted to the cochlea. Consequently, the comparatively poorer speech recognition observed in otosclerosis in relation to other middle ear pathologies with similar PTA values might reflect differences in mechanical sound transmission rather than the size of the air–bone gap alone. This underlines the importance to focus on more than PTA values in the evaluation and comparison of middle ear diseases with considerable CHL.

The effect of differing causes for CHL (stiffening, loss of compliance, increase of mass etc.) and MHL on speech understanding and hearing-related QoL is, to our best knowledge, underrepresented in literature.

Inferior WRS in MHL compared to CHL, relative to PTA, has been previously reported. Smith et al., in a large-scale analysis of speech-in-quiet performance, found that CHL patients generally performed better than those with MHL or sensorineural hearing loss, likely due to intact inner ear processing in CHL [10]. However, their study did not examine frequency-specific PTA correlations or disease-specific audiometric profiles. Our work addresses this gap by systematically evaluating multiple PTA calculation methods in relation to speech performance across distinct middle ear disorders. In prior research, we also emphasized the limitations of relying solely on AC, BC, and ABG closure in otosclerosis and advocated for a more nuanced approach to outcome assessment [22].

The AAO-HNS guidelines were revised in 2012 to include all types of hearing loss and recommended both PTA and WRS as minimum reporting standards. Our study reinforces the importance of a more nuanced approach, especially for MHL, and suggests that the choice between using 3 or 4 kHz in PTA calculation is of relatively minor importance when compared to broader disease-specific factors. It also emphasizes the need for broader adoption of standardized reporting that captures the complexity of hearing disorders beyond CHL.

Considering PROMs – and acknowledging the instrument-dependent nature of the commonly used and non-disease-specific SSQ12 as a generic hearing related measure not tailored to individual hearing conditions - we found that 4-frequency AC PTAs - regardless of whether they included 3 or 4 kHz - correlated significantly with subjective QoL as measured by the SSQ12, including most subscales except spatial hearing. In contrast, extended PTA calculations showed weaker correlations. Neither ABG-based PTAs nor speech audiometry demonstrated significant associations with SSQ12 scores. These findings suggest that AC-based PTA more accurately reflects real-world hearing impairment than conventional speech tests in quiet. The missing correlation for the subscale spatial hearing of the SSQ12 is probably related to the particularly demanding hearing situation represented in this subscale that did seem to be not sufficiently measured by PTA nor speech audiometry in quiet. These results suggest that AC-based PTA may reflect aspects of real-world hearing impairment captured by the SSQ12. However, this observation should be interpreted cautiously, given the small prospective cohort (*n* = 26) and the use of the SSQ12 as a generic rather than disease-specific QoL instrument. The observed associations may be influenced by sample size or questionnaire characteristics rather than representing a robust difference between audiometric measures. The lack of correlation between ABG-based PTAs and QoL may also have a conceptual explanation: patients likely perceive their absolute hearing ability (air conduction thresholds) as their primary handicap, whereas the air–bone gap primarily reflects the conductive component and potential surgical correctability rather than the experienced hearing difficulty itself. Therefore, using the SSQ12 is a limitation in the study design but using disease-specific questionnaires such as the COMOT-15 for COM would probably bias correlations between hearing tests and quality of life when different middle ear diseases including COM are compared.

Although our prospective cohort was small (*n* = 26), limiting statistical power especially for QoL analyses, we decided to include this cohort because of its high individual data quality and the opportunity to explore potential associations between hearing measures and patient-reported outcomes. We observed a trend toward correlation between 65 dB SPL speech scores and QoL for this cohort, whereas no such trend was seen for 50% number thresholds nor 80 dB SPL scores (Table 4). Measuring 50 dB SPL may have been even more effective but has not been done in our cohort with predominant CHL and overall rather mild hearing loss. For this reason, the 80 dB SPL scores did not differentiate and correlate in this cohort, because they were mostly 100% understanding and results are therefore not shown.

Despite the routine use of speech audiometry, our results show that it often correlates poorly with QoL - particularly at high levels like 80 dB SPL - which may not reflect the complexities of everyday listening. The discrepancy between objective test results and subjective experience further underscores the value of PTA-based metrics in evaluating hearing in CHL and MHL.

Supporting this, Dejaco et al. reported in a prospective study comparing hearing aids and active middle ear implants in otosclerosis that, despite audiometric and speech improvements, no consistent differences in hearing-related QoL (measured via the NCIQ) were observed between treatment groups [29]. This aligns with our finding that audiometric improvements do not necessarily translate into improved subjective hearing experience. Other QoL-studies in middle ear disease have predominantly focused on surgical outcomes, often failing to differentiate between speech tests and PTA when interpreting subjective results [30]. Comprehensive evaluations combining objective audiometric data with validated QoL instruments like the SSQ12 remain scarce.

## Limitations

The results of this study seem clinically relevant although caution is needed when interpreting isolated p-values due to our study design.

Given the number of statistical comparisons across different PTA configurations, outcome measures, and middle ear pathologies, the risk of type I error due to multiple testing must be considered. As the analyses were primarily exploratory and aimed at identifying general patterns rather than testing a single predefined hypothesis, no formal correction for multiple comparisons was applied. Therefore, individual p-values should be interpreted with caution and primarily in the context of the consistent patterns observed across analyses.

Our prospective cohort was small, which may have reduced statistical power, especially in subgroup and QoL analyses. While retrospective cohorts were larger, they were subject to common limitations such as incomplete documentation, cohort heterogeneity and selection bias. The combined retrospective/prospective (ambispective) study design further limits drawing statistically significant conclusions although collecting outcome parameters using a one-time measure with unchanged measurement set-up (excluding the additional collection of PROMs in the prospective cohort) minimizes potential bias when using an ambispective study design. Missing data for PTA as well as speech audiometry were imputated as described above. Although imputating missing data for PTA and speech audiometry is well established and the expected error minor, this method still entails a risk for bias [12, 13]. QoL assessment relied solely on the SSQ12, a brief but general tool not tailored to middle ear conditions. Disease-specific instruments like SPOT-25 (otosclerosis) or COMOT-15 (chronic otitis media) may have yielded more nuanced insights in the disease-specific cases [31, 32]. Alternative speech testing modalities - such as speech-in-noise or adaptive speech audiometry, may better reflect real-world deficits. Lastly, we did not adjust for potential confounders such as age, duration of disease, hearing aid use, cognitive status, or comorbidities, all of which may affect audiometric and subjective outcomes. Future studies should incorporate multivariate models to address these factors.

## Conclusion

We demonstrate strong correlations between various PTA calculation methods and speech recognition outcomes for both monosyllables and numbers. We found no clinically relevant difference between 4-frequency PTAs using 3 kHz or 4 kHz. However, we observed clinically significant differences in PTA-speech correlations between otosclerosis and otitis media cases, underlining the need for a more nuanced approach in reporting. Although speech audiometry retains its diagnostic role, it does not reliably correlate with QoL outcomes, highlighting the importance of incorporating PTA and validated QoL instruments in comprehensive patient evaluation.

In summary, there is little systematic, disease-specific research evaluating how different PTA strategies relate to both speech understanding and QoL across diverse middle ear conditions. Our study offers novel insights into this underexplored area, highlighting the importance of aligning audiometric assessment with both speech perception and patient experience in conductive and mixed hearing disorders, based on a real-world cohort with diverse middle ear pathologies. This aligns with the 2012 AAO-HNS guideline that has been broadened from CHL to any kind of hearing loss while at the same time adding WRS to PTA as minimal reporting standard.

## Supplementary Information

Below is the link to the electronic supplementary material.

Supplementary file1 (DOCX 21 KB)
