# Supplementary material for: Disease-specificity in correlations between speech audiometry, hearing-related quality of life, and varying calculation methods for pure tone average in middle ear diseases
Source: Eur Arch Otorhinolaryngol. 2026 Jun 1;283(9):5639–49. doi: 10.1007/s00405-026-10335-4 (PMC13615081; doi:10.1007/s00405-026-10335-4)
Supplement: Supplementary file 1 [file 405_2026_10335_MOESM1_ESM.docx]

Disease-specificity in correlations between speech audiometry, hearing-related quality of life, and varying calculation methods for pure tone average in middle ear diseases

**Supplemental material**

1. **Methods - Detailed case selection**
2. **Results - Supplemental Table S1**
1. **Methods - Detailed case selection**

Retrospective cases:

To screen for retrospective cases between 01/2013 and 03/2024, we identified 1,409 cases of otosclerosis (ICD H80.- (H80.0, H80.1, H80.2, H80.8, H80.9)) and, in total, 18,505 cases with possible AOM or COM (ICD H65.- (H65.0 - H65.9), H66.- (H66.0 - H66.9), H71, H72.- (H72.0 - H72.9), and H74.- (H74.0 - H74.9)). Subsequently, we screened cases in a reverse chronological order and tried to achieve almost balanced cohorts of 100 eligible ears each for our exploratory approach. Eligibility criteria were 1. adult age (≥18a at the time of the hearing test), 2. ENT-specialist proven diagnosis of a) otosclerosis, b) AOM or c) COM on one or both ears, 3. no previous middle ear surgery (except for myringotomy and/or grommet insertion), 4. available data on both pure tone and speech audiometry, 5. Normal or moderate BC (≤30 dBHL in every tested frequency), and 6. CHL of ≥10 dB in at least three different tested frequencies.

During the screening process we were able to identify 90 otosclerosis ears and would have been able to identify more than 90 ears with COM, but unfortunately only 40 ears with AOM. All AOM cases were measured in an acute phase of the disease, usually with at least 1 or 2 days distance to the onset of symptoms. In a further step, we re-investigated all clinical files of the 220 ears again and excluded further cases/ears (14 in the otosclerosis cohort, 8 in the AOM cohort and 4 in the COM cohort).

Prospective cases:

Between 2023 and 2025, a mixed prospective cohort was created using the above-defined eligibility criteria with patients willing to take a standardized tone and speech audiometry protocol as well as the SSQ12-QoL questionnaire. No disease-specific subcohorts were built because of small patient numbers. Most patients were ineligible because of the bone conduction or conductive hearing loss criterion, followed by prior middle ear surgeries such as tympanoplasty or stapes surgery, and minor age. Additionally, no acute cases are regularly seen in the specialized otology outpatient clinic.

2. **Results - Supplemental Table S1**

**Supplemental Table S1: Correlation between hearing test results and speech (linear regression)**

|  | **65dB monosyllables** | **50% number recognition** |
| --- | --- | --- |
| **cohort 1 (prospective)** |  |  |
| air conduction 0.5,1,2,3 kHz | R^2^=0.704 p<0.001 | R^2^=0.523 p<0.001 |
| air conduction 0.5,1,2,4 kHz | R^2^=0.673 p<0.001 | R^2^=0.498 p<0.001 |
| air conduction 0.5,1,2,3,4 kHz | R^2^=0.627 p<0.001 | R^2^=0.455 p<0.001 |
| 0.125,0.25,0.5,1,2,3,4,6,8 kHz | R^2^=0.523 p<0.001 | R^2^=0.284 p=0.005 |
| air bone gap 0.5,1,2,3 kHz | R^2^=0.493 p<0.001 | R^2^=0.321 p=0.003 |
| air bone gap 0.5,1,2,4 kHz | R^2^=0.506 p<0.001 | R^2^=0.344 p=0.002 |
| air bone gap 0.5,1,2,3,4 kHz | R^2^=0.471 p<0.001 | R^2^=0.318 p=0.003 |
| 0.25,0.5,1,2,3,4,6 kHz | R^2^=0.479 p<0.001 | R^2^=0.302 p=0.004 |
| **cohort 2 (otosclerosis)** |  |  |
| air conduction 0.5,1,2,3 kHz | R^2^=0.429 p<0.001 | R^2^=0.714 p<0.001 |
| air conduction 0.5,1,2,4 kHz | R^2^=0.445 p<0.001 | R^2^=0.714 p<0.001 |
| air conduction 0.5,1,2,3,4 kHz | R^2^=0.470 p<0.001 | R^2^=0.701 p<0.001 |
| 0.125,0.25,0.5,1,2,3,4,6,8 kHz | R^2^=0.436 p<0.001 | R^2^=0.675 p<0.001 |
| air bone gap 0.5,1,2,3 kHz | R^2^=0.243 p<0.001 | R^2^=0.536 p<0.001 |
| air bone gap 0.5,1,2,4 kHz | R^2^=0.320 p<0.001 | R^2^=0.565 p<0.001 |
| air bone gap 0.5,1,2,3,4 kHz | R^2^=0.292 p<0.001 | R^2^=0.550 p<0.001 |
| 0.25,0.5,1,2,3,4,6 kHz | R^2^=0.282 p<0.001 | R^2^=0.558 p<0.001 |
| **cohort 3 (AOM)** |  |  |
| air conduction 0.5,1,2,3 kHz | R^2^=0.204 p=0.016 | R^2^=0.732 p<0.001 |
| air conduction 0.5,1,2,4 kHz | R^2^=0.235 p=0.009 | R^2^=0.767 p<0.001 |
| air conduction 0.5,1,2,3,4 kHz | R^2^=0.214 p=0.013 | R^2^=0.708 p<0.001 |
| 0.125,0.25,0.5,1,2,3,4,6,8 kHz | R^2^=0.195 p=0.019 | R^2^=0.714 p<0.001 |
| air bone gap 0.5,1,2,3 kHz | R^2^=0.152 p=0.041 | R^2^=0.322 p=0.002 |
| air bone gap 0.5,1,2,4 kHz | R^2^=0.200 p=0.017 | R^2^=0.365 p<0.001 |
| air bone gap 0.5,1,2,3,4 kHz | R^2^=0.131 p=0.058 | R^2^=0.301 p=0.003 |
| 0.25,0.5,1,2,3,4,6 kHz | R^2^=0.174 p=0.027 | R^2^=0.327 p=0.001 |
| **cohort 4 (COM)** |  |  |
| air conduction 0.5,1,2,3 kHz | R^2^=0.291 p<0.001 | R^2^=0.572 p<0.001 |
| air conduction 0.5,1,2,4 kHz | R^2^=0.303 p<0.001 | R^2^=0.518 p<0.001 |
| air conduction 0.5,1,2,3,4 kHz | R^2^=0.275 p<0.001 | R^2^=0.473 p<0.001 |
| 0.125,0.25,0.5,1,2,3,4,6,8 kHz | R^2^=0.222 p<0.001 | R^2^=0.337 p<0.001 |
| air bone gap 0.5,1,2,3 kHz | R^2^=0.098 p=0.009 | R^2^=0.379 p<0.001 |
| air bone gap 0.5,1,2,4 kHz | R^2^=0.101 p=0.008 | R^2^=0.386 p<0.001 |
| air bone gap 0.5,1,2,3,4 kHz | R^2^=0.097 p=0.009 | R^2^=0.357 p<0.001 |
| 0.25,0.5,1,2,3,4,6 kHz | R^2^=0.125 p=0.003 | R^2^=0.346 p<0.001 |

*kHz=kilohertz; dB=decibels; AOM=acute otitis media; COM=chronic otitis media*
